# Broad host range of SARS-CoV-2 and the molecular basis for SARS-CoV-2 binding to cat ACE2

**DOI:** 10.1038/s41421-020-00210-9

**Published:** 2020-09-29

**Authors:** Lili Wu, Qian Chen, Kefang Liu, Jia Wang, Pengcheng Han, Yanfang Zhang, Yu Hu, Yumin Meng, Xiaoqian Pan, Chengpeng Qiao, Siyu Tian, Pei Du, Hao Song, Weifeng Shi, Jianxun Qi, Hong-Wei Wang, Jinghua Yan, George Fu Gao, Qihui Wang

**Affiliations:** 1 CAS Key Laboratory of Microbial Physiological and Metabolic Engineering, Institute of Microbiology, Chinese Academy of Sciences, Beijing, 100101 China; 2 University of Chinese Academy of Sciences, Beijing, 100049 China; 3 Institute of Physical Science and Information, Anhui University, Hefei, Anhui 230039 China; 4 CAS Key Laboratory of Pathogenic Microbiology and Immunology, Institute of Microbiology, Chinese Academy of Sciences, Beijing, 100101 China; 5 Faculty of Health Sciences, University of Macau, Macau, SAR China; 6 Ministry of Education Key Laboratory of Protein Sciences, Tsinghua-Peking Joint Center for Life Sciences, Beijing Advanced Innovation Center for Structural Biology, Beijing Frontier Research Center of Biological Structures, School of Life Sciences, Tsinghua University, Beijing, 100084 China; 7 Department of biomedical engineering, Emory University, Atlanta, GA 10033 USA; 8 Laboratory of Protein Engineering and Vaccines,Tianjin Institute of Industrial Biotechnology, Chinese Academy of Sciences, Tianjin, 300308 China; 9 School of Life Sciences, University of Science and Technology of China, Hefei, Anhui 230026 China; 10 Research Network of Immunity and Health (RNIH), Beijing Institute of Life Science, Chinese Academy of Sciences, Beijing, 100101 China; 11 Key Laboratory of Etiology and Epidemiology of Emerging Infectious Diseases in Universities of Shandong, Shandong First Medical University & Shandong Academy of Medical Sciences, Taian, Shandong 27100 China; 12 Savaid Medical School, University of Chinese Academy of Sciences, Beijing, 100049 China

**Keywords:** Cryoelectron microscopy, Molecular biology

## Abstract

Severe acute respiratory syndrome coronavirus 2 (SARS-CoV-2), the causative agent of the recent pandemic COVID-19, is reported to have originated from bats, with its intermediate host unknown to date. Here, we screened 26 animal counterparts of the human ACE2 (hACE2), the receptor for SARS-CoV-2 and SARS-CoV, and found that the ACE2s from various species, including pets, domestic animals and multiple wild animals, could bind to SARS-CoV-2 receptor binding domain (RBD) and facilitate the transduction of SARS-CoV-2 pseudovirus. Comparing to SARS-CoV-2, SARS-CoV seems to have a slightly wider range in choosing its receptor. We further resolved the cryo-electron microscopy (cryo-EM) structure of the cat ACE2 (cACE2) in complex with the SARS-CoV-2 RBD at a resolution of 3 Å, revealing similar binding mode as hACE2 to the SARS-CoV-2 RBD. These results shed light on pursuing the intermediate host of SARS-CoV-2 and highlight the necessity of monitoring susceptible hosts to prevent further outbreaks.

## Introduction

Emerging and re-emerging pathogens are a great threat to global public health^1^ and have caused tremendous economic loss, exemplified by the influenza virus in 1918 and highlighted by the recent coronavirus disease 2019 (COVID-19). The causative agent of COVID-19 was determined to be a novel coronavirus (CoV) and named as severe acute respiratory syndrome coronavirus 2 (SARS-CoV-2) by the International Committee on Taxonomy of Viruses (ICTV)^2^, in spite of some scientists proposing that HCoV-19 is more appropriate^3^. As of 24 August 2020, according to the World Health Organization (WHO), there are >23,000,000 confirmed cases and >800,000 related deaths in 216 countries (https://www.who.int/). Currently, no licensed therapeutics or vaccines are available yet. However, multiple vaccine candidates and therapeutic antibodies have entered into clinical trials^4,5^.

SARS-CoV-2 is the seventh coronavirus that could infect human beings^6,7^. CoVs are a group of enveloped viruses and contain a positive-sense and single-stranded RNA genome^8^. CoVs are categorized into four genera, namely alpha, beta, gamma and deltaCoVs (https://talk.ictvonline.org/). Two alphaCoVs (HCoV-NL63 and HCoV-229E), as well as two betaCoVs (HCoV-OC43 and HKU1), are the cause of common cold-like illnesses^9^. While three betaCoVs, namely SARS-CoV, Middle East respiratory syndrome coronavirus (MERS-CoV) and SARS-CoV-2, have all led to either epidemic or pandemic^7,10–12^.

Bats are identified as the natural reservoirs of a wide range of viruses including CoVs and play important roles in the transmission of these viruses. HCoV-NL63 was predicted to share common ancestry with an alphaCoV detected in the North American tricolored bat (*Perimyotis subflavus*)^13^. HCoV-229E has been reported to be highly related to CoVs carried by hipposiderid bats (*Hipposideros cf. ruber* or *Hipposideros abae*) in Africa^14^. Further genome comparison with alpaca CoVs revealed that alpacas seem to be the first host switched from bats, followed by a second interhost transfer from alpacas to humans^14,15^. Current evidence indicates that SARS-CoV originated from Chinese horseshoe bat (*Rhinolophus sinicus*) and subsequently transmitted to human directly or through civets^16^. MERS-CoV is also closely related to bat CoVs, with dromedary camels as a possible intermediate host as revealed by serological investigation^17^.

Several studies suggested that SARS-CoV-2 also originated from bat based on phylogenetic analysis. RaTG13, a bat CoV carried by a horseshoe bat (*Rhinolophus affinis*), shared the highest sequence identity (96.2%) to SARS-CoV-2^6^. RmYN02, a bat CoV detected in the Malayan horseshoe bat (*Rhinolophus malayanus*), displayed 93.3% identity to SARS-CoV-2^18^. In addition, RmYN02 contains three amino acid residues insertion at S1/S2 cleavage site of the spike (S) protein, which is similar to SARS-CoV-2, providing the evidence that SARS-CoV-2 may originate from recombination of bat CoVs.

Meanwhile, researchers are also making great efforts on investigating the intermediate host of SARS-CoV-2. Mink is predicted to be one reservoir candidate of SARS-CoV-2 by a virus host prediction (VHP) method based on deep learning algorithm^19^. Since the isolation of pangolin CoVs with high sequence similarity with SARS-CoV-2, pangolins are also believed to be potential intermediate hosts^20,21^. In addition, cats and ferrets are permissive to SARS-CoV-2 infection and cats experimentally transmit SARS-CoV-2 to naïve cats^22^. Notably, 14.7% (15/102) cat sera collected after the COVID-19 outbreak in Wuhan were positive for the SARS-CoV-2, while 39 cat sera collected prior to the outbreak are negative, demonstrating that SARS-CoV-2 infected the cat population in Wuhan during the outbreak^23^. Despite these reports and suspicions, the real intermediate host for SARS-CoV-2 remains elusive.

Virus infections start with the viral particles binding to the receptors on host cell surface. Consequently, for a virus to transmit to a new species, the gain-of-ability to bind to the cognate receptor of the target species is a prerequisite. CoVs utilize the S1 subdomain in S protein on the envelope to recognize the receptor. After the characterization of SARS-CoV-2, we and other researchers have reported that the C-terminal domain (CTD) in S1 of SARS-CoV-2 functions as a receptor binding domain (RBD) and specifically interacts with the angiotensin converting enzyme 2 (ACE2) protein that also serves as the receptor for SARS-CoV^24–26^. Therefore, characterizing the binding between SARS-CoV-2 RBD and ACE2 orthologs from various species could narrow down the potential intermediate hosts to the species with the ACE2s that interact with SARS-CoV-2 RBD.

Here, we chose 26 animals from 10 orders in Mammalia class, and chicken under the Galliformes order of Aves class, to analyze the functions and structures of the bindings between these ACE2 orthologs from potential intermediate host candidates and the RBD of SARS-CoV or SARS-CoV-2. Furthermore, we resolved the cryo-electron microscopy (cryo-EM) structure of cat ACE2 (cACE2) in complex with SARS-CoV-2 RBD at a resolution of 3 Å, and discovered that cACE2 utilizes a similar binding mode to interact with SARS-CoV-2 RBD comparing to human ACE2 (hACE2). The results in this study illustrated the broad range of species whose ACE2s could bind to SARS-CoV-2, including pets, domestic animals, and certain wild animals. We believe this research could shed light on the pursuit of the intermediate host candidates of the virus.

## Results

### Phylogenetic analysis of 26 animals based on ACE2 orthologs and characteristics of SARS-CoV-2 RBD-binding residues of ACE2s

**Fig. 1:**
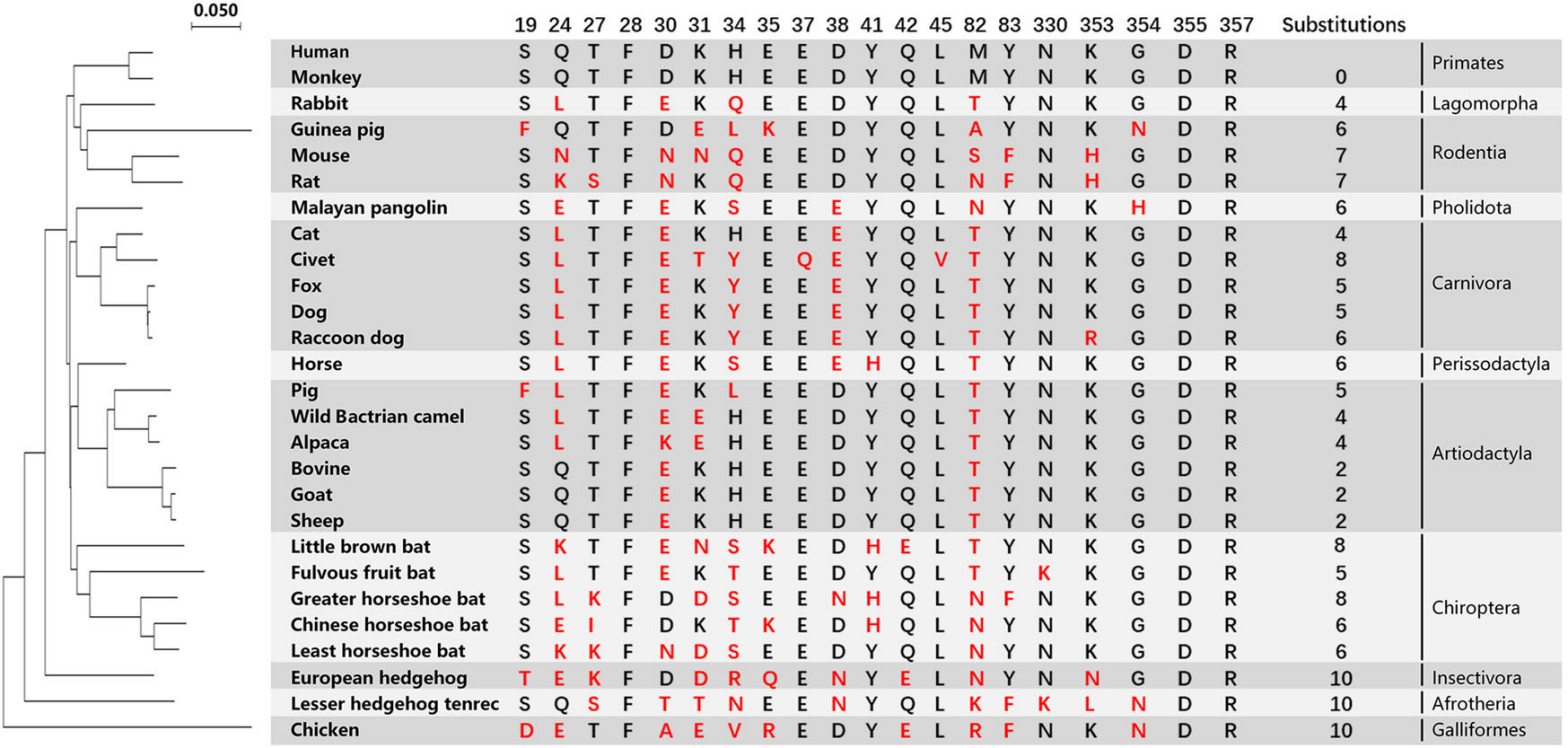
Phylogenetic analysis of 26 animals based on ACE2 and characteristics of the SARS-CoV-2 RBD-binding residues of ACE2s. Phylogenetic tree based on ACE2 amino acid sequences was generated using MEGA X. The 27 species (including human) belonging to 11 orders are shown in the right column. 20 residues of hACE2 which are crucial in interacting with the SARS-CoV-2 RBD are listed. Red letters indicate the substitutions in the ACE2 of 26 animal species.

To investigate the potential intermediate hosts and evaluate their possibility of being infected by SARS-CoV-2, we chose 26 animals, covering most domestic animals and companion pets, as well as some wild animals. For example, we include five bats and pangolin due to the previously reported detection or isolation of CoVs^27,28^. These 26 animals belong to 11 orders, including Primates, Lagomorpha, Rodentia, Pholidota, Carnivora, Perissodactyla, Artiodactyla, Chiroptera, Insectivora, Afrotheria, and Galliformes (Fig. 1). Based on the amino acid sequences of ACE2s, we constructed phylogenetic tree that showed the genetic relationship of 26 animals and human (Fig. 1). Among these animals, monkey and chicken showed the closest and farthest evolutionary distance relative to human, with the amino acid sequence identities of 95.16% and 66.62%, respectively. The other orthologs displayed identities with hACE2 ranging from 75.31% (lesser hedgehog tenrec) to 86.83% (horse).

The 20 key residues in hACE2 that are responsible for the interaction with SARS-CoV-2 RBD were highlighted and compared with 26 ACE2 orthologs. We found that, comparing to hACE2, the number of residue substitutions of ACE2 orthologs ranged from 0 to 10. Notably, the SARS-CoV-2 RBD-binding residues of monkey ACE2 are identical to that of hACE2, and European hedgehog, lesser hedgehog tenrec, and chicken had the most residue substitutions (10 for each). We therefore speculated that monkey is susceptible to SARS-CoV-2 similar to human, and European hedgehog, lesser hedgehog tenrec, and chicken seem to be unsusceptible to SARS-CoV-2.

From the residue comparison of ACE2s, we also found that the F28, D355, and R357 sites were completely conserved among these 27 species (Fig. 1 and Supplementary Fig. S1), and F28 seems to interact with F83/Y83, forming hydrophobic interaction and likely contributing to the stability of the two N-terminal helixes of ACE2s (Supplementary Figs. S1 and S2a, b). Civet ACE2 exclusively contains residues E37 and L45 substitutions (Fig. 1 and Supplementary Fig. S3). Residues equivalent to hACE2 Q24, D30, H34, and M82 were most diverse, with ACE2s of over 19 animals showing substitutions.

### Flow cytometric characterization of binding between ACE2 orthologs and the RBD of SARS-CoV-2 or SARS-CoV

**Fig. 2:**
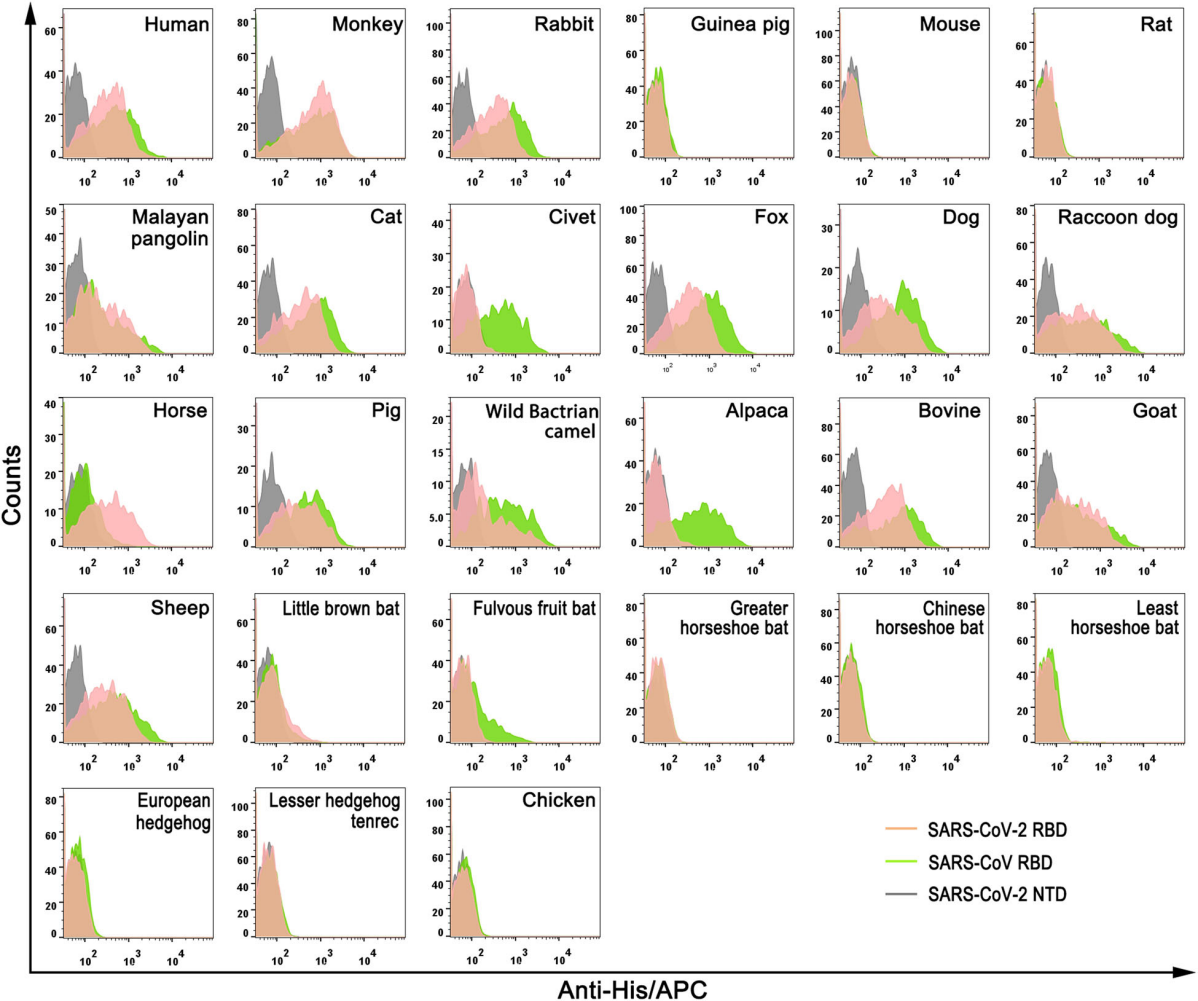
Flow cytometric characterization of the binding between ACE2s and SARS-CoV-2 RBD or SARS-CoV RBD. His-tagged SARS-CoV-2 RBD, SARS-CoV RBD and SARS-CoV-2 NTD proteins were incubated with HEK293T cells expressing eGFP-tagged ACE2s, respectively. Anti-His/APC antibody was used to detect the His-tagged protein binding to the cells. Cells stained with the SARS-CoV-2 RBD, the SARS-CoV RBD and the SARS-CoV-2 NTD proteins are shown in pink, brightgreen and gray, respectively. The SARS-CoV-2 NTD was used as the negative control.

We next tested the binding of SARS-CoV-2 RBD or SARS-CoV RBD protein to eGFP-fused ACE2s expressed on cell surface via flow cytometry (FACS). SARS-CoV-2 NTD protein was used as a negative control. As illustrated in Fig. 2, SARS-CoV-2 RBD evidently interacted with the cells expressing ACE2 orthologs from animals that belong to Primates (monkey), Lagomorpha (rabbit), Pholidota (Malayan pangolin), Perissodactyla (horse), most Carnivora (cat, fox, dog, and raccoon dog) and most Artiodactyla (pig, wild Bactrian camel, bovine, goat and sheep), but not the ones from Rodentia (guinea pig, mouse, and rat), Insectivora (European hedgehog), Afrotheria (lesser hedgehog tenrec), or Galliformes (chicken). Notably, the ACE2 orthologs from five bat species under the *Eptesicus* (little brown bat), *Rousettus* (fulvous fruit bat) and *Rhinolophus* (greater horseshoe bat, Chinese horseshoe bat and least horseshoe bat) genera exhibited varieties, with the former two displaying minimal fluorescent shift due to the SARS-CoV-2 RBD binding and the latter three showed undetectable interaction.

SARS-CoV RBD displayed binding patterns similar to SARS-CoV-2 RBD, with the exception of civet and alpaca ACE2s. In contrast to the SARS-CoV-2 RBD, which displayed undetectable interaction with civet or alpaca ACE2 in FACS data, incubation of the SARS-CoV RBD lead to the overt fluorescent shift of cells with civet and alpaca ACE2s (Fig. 2). No cell interacts with the SARS-CoV-2 NTD as previously reported (Fig. 2)^24^.

Notably, previous studies indicated that the glycan moiety at the residue equivalent to hACE2 M82 would disrupt the interactions between ACE2 and SARS-CoV RBD^29^. As indicated in Supplementary Fig. S1, potential glycosylated N82 exists in the ACE2s of both rat and greater horseshoe bat, but not in Malayan pangolin (NYQ), Chinese horseshoe bat (NYP), least horseshoe bat (NYP) or European hedgehog (NYP). Thus, we introduced N82M to both rat ACE2 (rat ACE2-N82M) and greater horseshoe bat ACE2 (greater horseshoe bat ACE2-N82M) and detected their interactions with SARS-CoV-2 RBD or SARS-CoV RBD via FACS. Our results indicated that the two mutants displayed the same binding characteristic as their wild type counterparts, with no detectable binding with either SARS-CoV-2 RBD or SARS-CoV RBD (Supplementary Fig. S4).

### Surface plasmon resonance assays (SPR) characterization of specific interaction between ACE2 orthologs with the RBD of SARS-CoV-2 or SARS-CoV

**Fig. 3:**
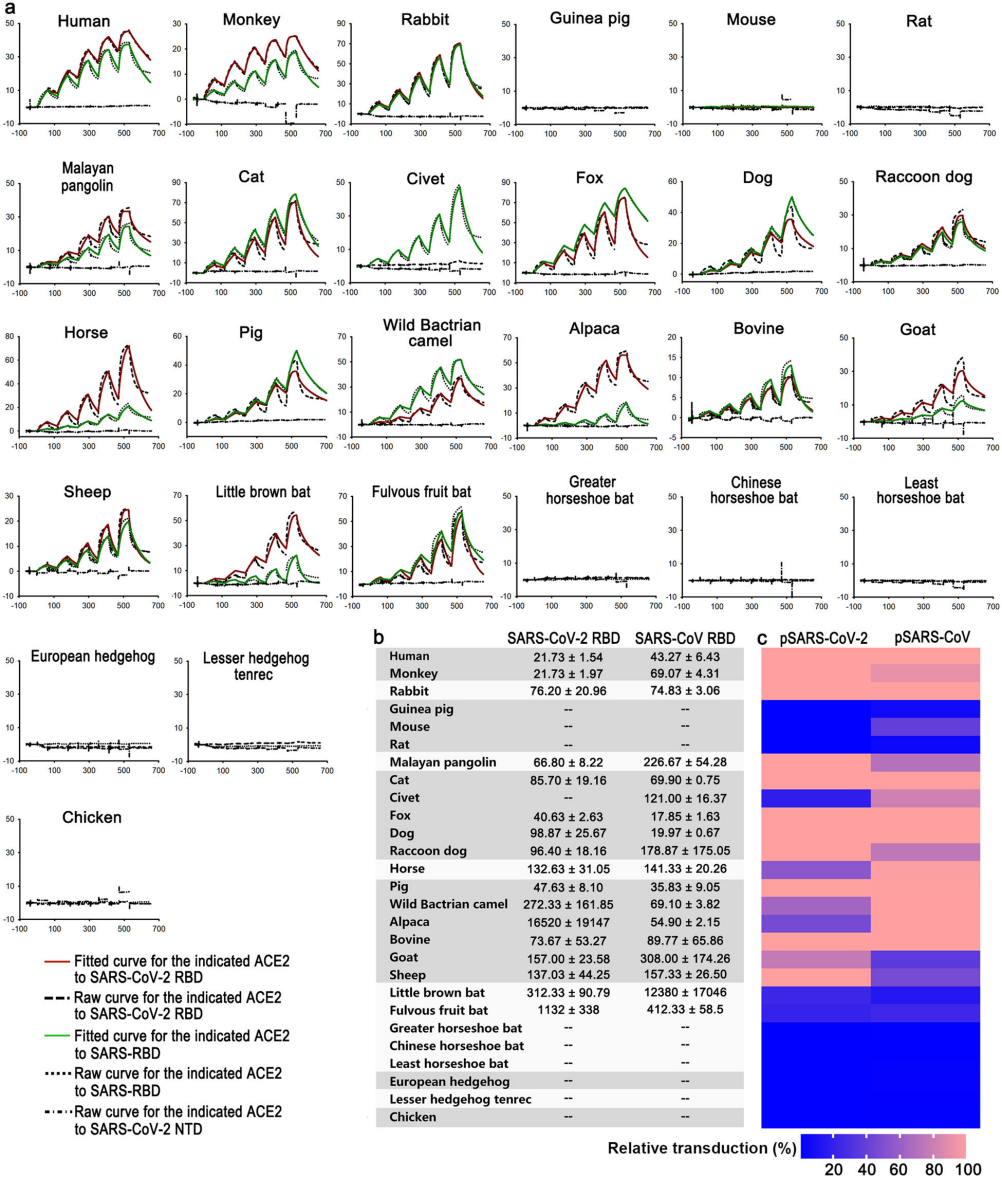
SPR characterization of the binding between ACE2s and SARS-CoV-2 RBD or SARS-CoV RBD, and ACE2s mediated pseudoviruses transduction. **a** The mFc-tagged ACE2s in supernatants were captured by anti-mIgG Fc antibodies immobilized on the CM5 chip, and sequentially tested the binding with serially diluted SARS-CoV-2 RBD or SARS-CoV RBD. The SARS-CoV-2 NTD was used as the negative control. The raw and fitted curves were displayed in dotted and solid lines, respectively. **b** The binding affinities between ACE2s and SARS-CoV-2 RBD or SARS-CoV RBD are shown with the means ± SD of three independent experiments. **c** BHK21 cells expressing the indicated ACE2 orthologs were infected with SARS-CoV-2 or SARS-CoV pseudovirus containing luciferase-reporter. Luciferase activity was determined at 24 h post infection. Relative transduction values (%) for each ACE2 ortholog mediated pseudovirus transduction were normalized to hACE2 and presented as a heatmap according to the indicated color code. Pseudovirus transduction were performed at least twice for each ACE2 with three replicates. Data shown are representative data with the mean of three replicates.

To better understand the interactions between 26 ACE2 orthologs and the RBD of SARS-CoV-2 or SARS-CoV, we determined the binding affinities via SPR. The mouse Fc (mFc)-tagged ACE2s were first captured by the chip pre-immobilized with anti-mouse IgG antibodies, and then the serially diluted SARS-CoV-2 RBD, SARS-CoV RBD or SARS-CoV-2 NTD proteins were flowed through the chip. Consistent with the FACS assay, SARS-CoV-2 RBD interacted with ACE2 orthologs from Primates, Lagomorpha, Pholidota, Perissodactyla, most Carnivora and most Artiodactyla with varied binding affinities (Fig. 3a, b). Specifically, the monkey ACE2 interacted with SARS-CoV-2 RBD with the same strength as hACE2. In comparison, the binding affinity of SARS-CoV-2 RBD to the fox and pig ACE2s were 2-fold weaker, the Malayan pangolin, bovine, rabbit, cat, dog and raccoon dog ACE2s were 3–4-fold weaker, and the horse, goat, and sheep ACE2s were 6–7-fold weaker. For the wild Bactrian camel, little brown bat, and fulvous fruit bat, their ACE2 orthologs were further weaker, with equilibrium dissociation constant (*K*_D_) increased by more than 10-fold (Fig. 3b). No binding between the civet ACE2 and the SARS-CoV-2 RBD was observed, which is similar to the corresponding FACS result (Figs. 2, 3). Although no interaction between the alpaca ACE2 and SARS-CoV-2 RBD by FACS was detected, the *K*_D_ for this pair of interaction was calculated to be 16.5 μM (Fig. 3a, b).

For the SARS-CoV RBD, most of the interactive affinities with ACE2 orthologs were similar to the level of the SARS-CoV-2 RBD interacting with the corresponding species, except for the interactions with the fox and dog ACE2s which were even stronger than the level of the SARS-CoV-2 RBD interacting with hACE2 (Fig. 3a, b). In contrast to the SARS-CoV-2 RBD, the SARS-CoV RBD also interacted with the civet and alpaca ACE2, and bound to the wild Bactrian camel ACE2 with fourfold higher affinity than that of SARS-CoV-2 RBD. In addition, the SARS-CoV RBD interacted with Malayan pangolin and little brown bat ACE2 with ~4-fold and 40-fold lower affinities than SARS-CoV-2 RBD, respectively. Though both SARS-CoV-2 and SARS-CoV were thought to have originated from bats, their interaction with the bat ACE2s in this study were relatively low (Fig. 3a, b).

### The transduction of pseudotyped SARS-CoV-2 or SARS-CoV engaged by ACE2s

With evidence of binding between ACE2 orthologs and the RBD of SARS-CoV-2 or SARS-CoV, we then tested the potential of these ACE2 orthologs functioning as the receptors for SARS-CoV-2 or SARS-CoV. Pseudoviruses, which incorporated the S protein of SARS-CoV-2 or SARS-CoV, also encode luciferase for determining the transduction efficiency via quantification of luciferase activities in the cell lysates. BHK21 cells, which are unsusceptible for SARS-CoV-2 and SARS-CoV, were transfected with the plasmids encoding each of 27 eGFP-tagged ACE2 orthologs. Then the eGFP-positive cells were sorted for evaluating the transduction of pseudoviruses. We found that the monkey, rabbit, Malayan pangolin, cat, fox, dog, raccoon dog, pig and bovine ACE2s supported pseudotyped SARS-CoV-2 transduction as good as hACE2, while the ACE2 orthologs from horse, wild Bactrian camel, alpaca as well as goat and sheep are less efficient than hACE2. Consistent with the binding affinities with SARS-CoV-2 RBD, the bat ACE2s, which could initiate the entry of SARS-CoV-2 pseudoviruses at a low level are from little brown bat and fulvous fruit bat, but not from greater horseshoe bat, Chinese horseshoe bat, or least horseshoe bat. Although the civet ACE2 displays no detectable binding with the SARS-CoV-2 RBD, it could still mediate the transduction of pseudotyped SARS-CoV-2 (Fig. 3 and Supplementary Fig. S5). The ACE2 orthologs from animals belonging to Rodentia, Insectivora, Afrotheria and Galliformes cannot support pseudotyped SARS-CoV-2 transduction (Fig. 3c).

Similarly, the SARS-CoV pseudovirus could efficiently enter cells expressing the ACE2 orthologs from rabbit, cat, fox, dog, pig and bovine, but not guinea pig, rat, European hedgehog, lesser hedgehog tenrec or chicken. The ACE2 orthologs of horse, wild Bactrian camel and alpaca could also efficiently mediated transduction of SARS-CoV pseudovirus at the level similar to hACE2. The monkey, mouse, Malayan pangolin, civet, raccoon dog, goat, and sheep ACE2s showed relatively low ability. Notably, although the bindings between the RBDs and ACE2 from little brown bat or fulvous fruit bat were weak, they still supported the transduction of both pseudoviruses. Consistent with the binding features, the ACE2s from three horseshoe bats were not observed to support either pseudoviruses transduction (Fig. 3c).

### Molecular basis of the interaction between the cACE2 and the SARS-CoV-2 RBD and the comparison with the complex of the SARS-CoV-2 RBD with hACE2

Currently, there are multiple evidences showing the susceptibility of cat to SARS-CoV-2 infection, including the experimental infection data, the serological study in Wuhan, as well as the binding characterizations in this study. To further elucidate the molecular basis of the cACE2 binding to SARS-CoV-2 RBD, we prepared the SARS-CoV-2 RBD-cACE2 complex by in vitro mixture of the two proteins and then purified via a gel filtration. The cryo-EM complex structure was solved at 3 Å resolution with one SARS-CoV-2 RBD binding to a single cACE2 molecule (Table 1).

**Table 1:** Cryo-EM data collection, refinement and validation statistics of cACE2 in complex with SARS-CoV-2 RBD.

	cACE2 in complex with SARS-CoV-2 RBD
Data collection and processing	
Magnification	130k
Voltage (kV)	300
Electron exposure (e^-^/Å^2^)	50
Defocus range (μm)	−1.8 to −2.2
Pixel size (Å)	0.99375
Symmetry imposed	C1
Final particle images (no.)	195,370
Map resolution (Å)	3.0
FSC threshold	0.143
Refinement	
Initial model used (PDB code)	6LZG
Model resolution range (Å)	up to 3
FSC average (model to map)	
Whole unit cell	0.7298
Around atoms	0.74
Model composition	
Non-hydrogen atoms	6359
Protein residues	792
Ligands	1
* B* factors (Å^2^)	
Protein	55.1
Ligand	56.7
R.m.s. deviations	
Bond lengths (Å)	0.003
Bond angles (°)	0.481
Validation	
MolProbity score	2.29
Clashscore	8.71
Poor rotamers (%)	5.90
Ramachandran plot	
Favored (%)	96.45
Allowed (%)	3.55
Outliers (%)	0

**Fig. 4:**
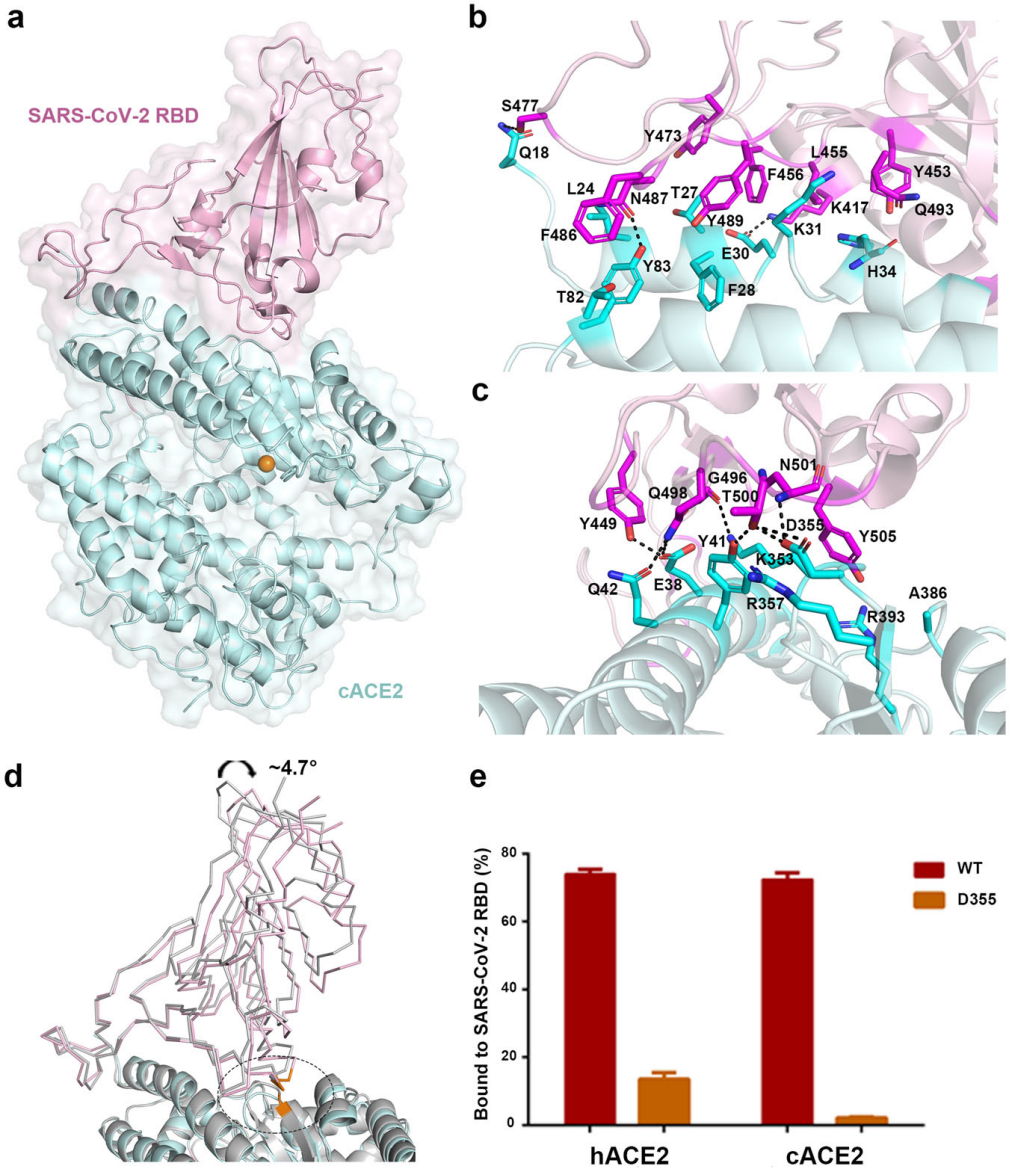
The complex structure between cACE2 and SARS-CoV-2 RBD. **a** The overall complex structure of cACE2 bound to SARS-CoV-2 RBD. cACE2 and SARS-CoV-2 RBD were colored in lightpink and palecyan, respectively. **b**, **c** The detailed interaction between cACE2 and the SARS-CoV-2 RBD. The residues involved in the interaction were labeled, and H-bonds were shown as dotted lines with a cutoff of 3.3 Å. **d** The overall comparison between the complex of cACE2 and SARS-CoV-2 RBD and that of hACE2 and SARS-CoV-2 RBD. hACE2 and SARS-CoV-2 RBD complex were colored in gray. SARS-CoV-2 RBDs and ACE2s were displayed in ribbon and cartoon, respectively. Residue D355 of ACE2 and interacted residues on SARS-CoV-2 RBD were circled, related to Supplementary Fig. S2c, d. **e** HEK293T cells transfected with pEGFP-N1-cACE2, hACE2 (WT), or the mutants containing D355A were incubated with His-tagged SARS-CoV-2 RBD protein. Anti-His/APC antibody was used to detect the His-tagged protein binding to the cells by flow cytometry. The percentage of the indicated ACE2-expressing cells that were bound to SARS-CoV-2 RBD were shown as a histogram. The assays were independently performed twice. One representative data was displayed with the mean of triplicates (*n* = 3), and the bar represented the SD value.

The overall structure with one SARS-CoV-2 RBD bound to one cACE2 molecule resembles the complex structure of the SARS-CoV-2 RBD binding with hACE2, with the root mean square deviation of 0.763 Å for 648 Cα atoms (Fig. 4a). To describe the detailed interaction, residues contributing to the van der Waals (vdw) interaction between the cACE2 and the SARS-CoV-2 RBD were listed in Table 2, with a cutoff of 4 Å, and residues involving in hydrogen bond (H-bond, with a cutoff of 3.3 Å) and salt bridge interactions were labeled (Fig. 4b, c). As the SARS-CoV-2 RBD interacting with hACE2, the interface between the SARS-CoV-2 RBD and the cACE2 also involves in H-bond and salt bridge interactions. Seven residues (L455, F456, Y473, S477, F486, N487, and Y489) on the β1′/β2′ loop of the SARS-CoV-2 RBD contributed 45 contacts with the cACE2, including two H-bonds. At the equivalent interface between the SARS-CoV-2 RBD and hACE2, eight residues (L455, F456, A475, G476, F486, N487, Y489, and F490) contributed 48 contacts, with three H-bonds (Table 2). Ten residues (G446, Y449, Y453, Q493, G496, Q498, T500, N501, G502, and Y505) on α1′/β1′ loop and β2′/η1′ loop of SARS-CoV-2 RBD formed 94 and 91 contacts to the cACE2 and hACE2, including eight H-bonds and nine H-bonds, respectively. Residue K417 of the SARS-CoV-2 RBD formed salt bridge interactions with residue E30 of cACE2, and D30 of hACE2 (Table 2). These results indicated when the SARS-CoV-2 RBD bound to the cACE2, the contribution of β1′/β2′ loop decreased but α1′/β1′ loop and β2′/η1′ loop increased, which resulted in a ~ 4.7° angle shift, while the N-terminal 85 residues of the cACE2 were superimposed with hACE2 (Fig. 4d).

**Table 2:** Comparison of SARS-CoV-2 RBD binding to cACE2 or hACE2.

SARS-CoV-2 RBD	cACE2	hACE2
K417 (4,1/3,1)	E30 (4,1)	D30 (3,1)
G446 (1/2,1)	Q42 (1)	Q42 (2,1)
Y449 (12,1/7,2)	E38 (9,1), Q42 (3)	D38 (5,1), Q42 (2,1)
Y453 (5/3,1)	H34 (5)	H34 (3,1)
L455 (5/4)	E30 (1), H34 (4)	H34 (4)
F456 (13/7)	T27 (4), E30 (4), K31 (5)	T27 (5), D30 (1), K31 (1)
Y473 (1/0)	T27 (1)	
A475 (0/5,1)		S19 (3,1), Q24 (1), T27 (1)
G476 (0/2)		S19 (2)
S477 (4,1/0)	Q18 (4,1)	
F486 (5/11)	T82 (5)	M82 (4), Y83 (7)
N487 (7,1/11,2)	L24 (3), Y83 (4,1)	Q24 (7,1), Y83 (4,1)
Y489 (10/7)	T27 (1), F28 (6), K31 (2), Y83 (1)	T27 (2), F28 (4), Y83 (1)
F490 (0/1)		K31 (1)
Q493 (7/6)	H34 (7)	H34 (3), E35 (3)
G496 (9,1/6,1)	E38 (3), K353 (6,1)	D38 (1), K353 (5,1)
Q498 (16,2/11,2)	E38 (6,1), Y41(7), Q42 (3, 1)	Y41(5), Q42(5,2), L45(1)
T500 (18, 3/18)	Y41 (3,1), D355 (12,2), R357 (3)	Y41 (6), N330 (3), D355 (6), R357 (3)
N501 (6,1/10,1)	Y41 (1), K353 (1), D355 (4,1)	Y41 (5,1), K353 (5)
G502 (5/8,1)	K353 (1), D355 (4)	K353 (3,1), G354 (5)
Y505 (15/19)	K353 (9), G354 (2), A386 (1), R393 (3)	E37 (3), K353 (14), G354 (2)
Total	143, 11	141, 13

The numbers in parentheses of SARS-CoV-2 RBD residues represent the number of vdw and H-bond contacts between the indicated residue with cACE2 (the former) and hACE2 (the latter). The numbers in parentheses of ACE2s residues represent the numbers of vdw contacts the indicated residues conferred. The numbers with underline suggest numbers of potential H-bonds between the pairs of residues. vdw contact was analyzed at a cutoff of 4 Å and H-bonds at a cutoff of 3.3 Å.

In particular, residue D355 of the cACE2 formed three H-bonds with the SARS-CoV-2 RBD, but hACE2 D355 formed vdw interaction with the SARS-CoV-2 RBD (Table 2 and Supplementary Fig. S2c, d). Consistently, the cACE2 D355A lost the binding to the SARS-CoV-2 RBD, while hACE2 carrying D355A maintained the partial interaction with the SARS-CoV-2 RBD (Fig. 4e). Though the total number of contacts of the SARS-CoV-2 RBD interacting to the cACE2 and hACE2 (143 and 141, respectively) were similar, the total number of H-bonds of the SARS-CoV-2 RBD and the cACE2 was less than that of the SARS-CoV-2 RBD and hACE2 (11 and 13, respectively), which agrees with the binding features to the SARS-CoV-2 RBD.

## Discussion

The spread of SARS-CoV-2 places the world in a global crisis. Investigating the source of this novel CoV is not only a scientific issue, but also a crucial matter for the control and prevention of related infectious diseases in human population^30^. Due to the complete disinfection of the wet market linked to this virus outbreak, such investigation has become extremely challenging, which desperately needs extensive screening of the wild animals, including virus detection and isolation, and serological studies. Fortunately, as an alternative method, clues for the tracking the origin of the virus can be found through characterizing the interaction between the SARS-CoV-2 S protein and the ACE2 orthologs from a broad range of species. The reason is that, to perform inter-species transmission, the gain-of-function to bind to the cell surface receptor of a different species is a prerequisite for the virus, and such gain-of-function leaves trails in the different binding affinity of the viral RBD to various host receptors.

We further evaluated the interaction between the SARS-CoV-2 RBD and 26 ACE2s and found that this RBD could interact with ACE2s from 17 species, including animals belonging to Primates (monkey), Lagomorpha (rabbit), Pholidota (Malayan pangolin), Carnivora (cat, civet, fox, dog, and raccoon dog), Perissodactyla (horse), Artiodactyla (pig, wild Bactrian camel, alpaca, bovine, goat, and sheep), and Chiroptera (little brown bat and fulvous fruit bat). Some animals could be excluded, including the selected animals belonging to Rodentia (guinea pig, mouse, and rat), Insectivora (European hedgehog), Afrotheria (lesser hedgehog tenrec), and Galliformes (chicken).

The glycosylation of ACE2 plays important role in the interactions between receptor and virus. In a previous study, the glycans linked to the residue equivalent to hACE2 M82 are hypothesized to disrupt the interactions between rat ACE2 and SARS-CoV RBD, based on structural analysis. However, our FACS results show that depletion of potential glycosylation at N82 in either rat or greater horseshoe bat ACE2 cannot change the binding characteristics of either ACE2 to SARS-CoV RBD or SARS-CoV-2 RBD (Supplementary Fig. S4). Thus, the substitutions of key residues, which are responsible for the interaction with the ligands in the receptors, should be the main reason for the loss of interactions.

The SARS-CoV RBD likely interacts with ACE2s from a broader range of hosts, including mouse. Although mouse ACE2 shows no detectable binding with SARS-CoV RBD through FACS and SPR, this receptor supports the entry of SARS-CoV pseudovirus into cells. Consistent with a recent report^31^, the civet ACE2 could mediated the transduction of SARS-CoV-2 pseudovirus at a much lower level than hACE2, in spite of showing no detectable binding with SARS-CoV-2 RBD (Supplementary Fig. S5). Civet was hypothesized to transmit SARS-CoV^16,27^. The different binding features of SARS-CoV and SARS-CoV-2 to orthologs of the same receptor indicate the two viruses have different transmission routes. Notably, civet exclusively contains E37Q and L45V, together with another six residue substitutions. According to the interaction between SARS-CoV-2 RBD and hACE2, the side chain of residue Y505 on SARS-CoV-2 RBD inserts into the groove related to E37 of hACE2 by electrostatic interaction (Supplementary Fig. S3). The substitution of E37 with glutamine shifts the electrostatic feature to the opposite and might contribute to the decreased ability of civet ACE2 to interact with SARS-CoV-2 RBD.

Multiple evidence supports the susceptibility of cats to SARS-CoV-2, including the cat samples in Wuhan collected after the outbreak circulating the antibodies against SARS-CoV-2^23,32^. In this study, the SARS-CoV-2 RBD is found to interact with the cACE2, albeit with a lower binding affinity than with hACE2. The cryo-EM structure of the cACE2 in complex with the SARS-CoV-2 RBD reveals that SARS-CoV-2 utilizes the similar binding mode to bind to both receptors, but forms more H-bonds with hACE2 than with the cACE2, which is consistent with the binding features. Whether cats are the intermediate host of SARS-CoV-2 needs further studies. One important question is whether the stray cats in Wuhan or the surrounding areas before the outbreak contain the antibodies against SARS-CoV-2. In addition, a tiger (another animal belonging to Felidae) in an American zoo was confirmed to be infected by SARS-CoV-2^33^, indicating more efforts are needed to study the role of felines in the transmission and evolution of SARS-CoV-2.

Bats are the natural reservoir of many viruses. Identification of RaTG13 and RmYN02 suggests that SARS-CoV-2 may have bat origin, but powerful evidence is still lacking^6,18^. Unlike the bat CD26s from multiple species that binds to the MERS-CoV RBD with varied binding affinities^34^, the SARS-CoV-2 RBD interacts with the ACE2s from little brown bat and fulvous fruit bat, but not the ones from the three horseshoe bats tested in this study. Recently, a paper submitted in bioRxiv reported the polymorphism of Chinese horseshoe bats, especially at the N-terminal region is responsible for the binding to SARS-CoV-2 and SARS-CoV^35^. Eight different ACE2s were detected in Chinese horseshoe bats. Even in one cave, Chinese horseshoe bats carry four different sequences. The Chinese horseshoe bat ACE2 in this study is the same as allele 8. The SARS-CoV RBD interacts with six out of the eight sequences, while allele 8 did not support viral entry, which is consistent with the results reported here. The diversity of ACE2 provides the selective pressure for the evolution of SARS-CoV-2, SARS-CoV and other ACE2-binding CoVs, highlighting the necessity for long-term surveillance of bat CoVs.

It seems that diversified ACE2s could support SARS-CoV-2 entry. However, after viruses enter the susceptible host or cells, hosts would mobilize the intercellular and intracellular immunity, with multiple host factor involved, to combat the viruses. Thus, the result for a virus infection would depend on the game between viruses and hosts. As indicated in this study, although the SARS-CoV-2 RBD binds to the dog ACE2 and the pig ACE2 with high affinities, SARS-CoV-2 replicates poorly in these two animals^22^. Thus, more studies, including viral challenge at the BSL-3 lab and SARS-CoV-2-specific antibody detections in the wild animals, are needed to further pursue the intermediate hosts of SARS-CoV-2. In addition, through evaluating the interactions between SARS-CoV-2 RBD and ACE2s from various animals, multiple species are found to have risk of being infected by SARS-CoV-2, and have the potential to become animal reservoirs for virus transmission, as exemplified by the recently reported mink^36^. In summary, our results provide directions for hunting intermediate hosts of SARS-CoV-2 and highlight the necessity of monitoring susceptible hosts to prevent further outbreaks.

## Materials and methods

### Gene cloning

The full-length ACE2 coding sequences of 26 animals (accession numbers are shown in Supplementary Table S1) were synthesized and respectively cloned into pEGFP-N1 vector used for flow cytometry. The ectodomains of the 26 ACE2s fused with the Fc domain of mouse IgG (mFc) were individually cloned into pCAGGS vector using *Eco*RI and *Xho*I restriction sites used for SPR.

The pFastBac plasmids expressing SARS-CoV-2 RBD (residues 319–541, GISAID: EPI_ISL_402119), SARS-CoV-2 NTD (residues 20–286, GISAID: EPI_ISL_402119) and SARS-CoV RBD (residues 306–527, GenBank: NC_004718) used for both flow cytometry and SPR were constructed in our previous work^24^.

The coding sequence of cACE2 (residues 18–740) was synthesized and cloned into pET21a vector (pET21a-cACE2) used for protein expression and purification.

### Protein expression and purification

The SARS-CoV-2 RBD, SARS-CoV-2 NTD, and SARS-CoV RBD proteins used for flow cytometry and SPR experiments were expressed and purified using Bac-to-Bac baculovirus expression system (Invitrogen) as described in our previous work^24^.

To prepare the mFc-tagged ACE2 proteins, the pCAGGS plasmids containing the coding sequences of ACE2s were transiently transfected into HEK293T cells. 48 h later, supernatant containing the indicated protein were collected, concentrated and then used for SPR assays.

The pET21a-cACE2 was transformed into *Escherichia coli* (*E. coli*) strain BL21 (DE3) for protein expression. cACE2 was over expressed in *E. coli* as inclusion bodies and refolded as previously^37^. Briefly, the dissolved cACE2 inclusion bodies were diluted dropwise in a refolding buffer (100 mM Tris-HCl, pH 8.0, 2 mM EDTA, 400 mM L-arginine, 0.5 mM oxidized glutathione and 5 mM reduced glutathione) at 4 °C overnight. The refolded cACE2 proteins were concentrated using an Amicon 8400 concentrator with 10 kDa cutoff membrane and changed into 20 mM Tris-HCl (pH 8.0) and 150 mM NaCl buffer and subsequently purified by gel-filtration chromatography with a HiLoad 16/600 SuperdexTM 200 pg column (GE Healthcare) using ÄKTA System.

To obtain the cACE2 and SARS-CoV-2 RBD complex, purified cACE2 and SARS-CoV-2 RBD proteins were mixed in a 1:2 molar ratio and incubated for 1 h on ice. The mixture was then purified with a HiLoad 16/600 SuperdexTM 200 pg column (GE Healthcare) in 20 mM Tris-HCl (pH 8.0) and 150 mM NaCl buffer. The complex peak of the cACE2 with the SARS-CoV-2 RBD was collected and concentrated to ~0.2 mg/mL for cryo-EM.

### Flow cytometry analysis

To test the binding between the ACE2s and SARS-CoV-2 RBD or SARS-CoV RBD, the 27 ACE2s fused with eGFP were expressed on the cell surface by transfecting each of the 27 pEGFP-N1-ACE2s plasmids into HEK293T cells using PEI (Alfa). In total, 6 h later, the cell culture was replaced with fresh DMEM with 10% FBS (Gibco). In total, 24 h post transfection, 2 × 105 cells were collected, resuspended in PBS and incubated with SARS-CoV-2 RBD, SARS-CoV RBD and SARS-CoV-2 NTD proteins at a concentration of 1 μg/mL at 37 °C for 30 min. Subsequently cells were washed twice with PBS and further stained with anti-His/APC antibody (1:500, Miltenyi Biotec) for another 30 min at 37 °C. After washing, the cells were analyzed using BD FACSCanto. The cells transfected with pEGFP-N1-hACE2 were used as positive control. To evaluate the binding between SARS-CoV-2 RBD and cACE2 (WT), hACE2 (WT) or mutants containing D355A, we expressed GFP-tagged cACE2, hACE2, or the mutants on the cell surface, and then stained the cells with His-tagged SARS-CoV-2 RBD protein. Anti-His/APC antibody was used to detect the His-tagged protein binding to the cells. The percentage of the indicated ACE2-expressing cells that were bound to SARS-CoV-2 RBD were shown as a histogram. The assays were independently performed twice. One representative data displayed in Fig. 4e was the mean of triplicates (*n* = 3), and the bar represented the SD value.

### SPR analysis

We tested the binding affinities between the mFc-tagged ACE2s and SARS-CoV-2 RBD or SARS-CoV RBD proteins by SPR using a BIAcore 8K (GE Healthcare) carried out at 25 °C in single-cycle mode. SARS-CoV-2 NTD protein was used as negative control. The HBS-EP buffer (20 mM HEPES, pH 7.4, 150 mM NaCl, and 0.005% (v/v) Tween 20) was used as the running buffer, and SARS-CoV-2 RBD, SARS-CoV RBD and SARS-CoV-2 NTD proteins were changed into this buffer by gel filtration before use. First, the anti-mFc antibodies were immobilized on the CM5 biosensor chip (GE Healthcare) using amine-coupling chemistry protocol (GE Healthcare). Then, the supernatants containing mFc-tagged ACE2s were injected and captured respectively at ~100–700 response units. SARS-CoV-2 RBD, SARS-CoV RBD or SARS-CoV-2 NTD protein was serially diluted and flowed through the chip surface and the binding response was measured. Briefly, 100, 50, 25, 12.5, and 6.25 nM of SARS-CoV-2 RBD, SARS-CoV RBD, or SARS-CoV-2 NTD protein were used to test the binding to dog or pig ACE2. 200, 100, 50, 25, and 12.5 nM of SARS-CoV-2 RBD, SARS-CoV RBD or SARS-CoV-2 NTD protein were used to guinea pig, civet, greater horseshoe bat, Chinese horseshoe bat, least horseshoe bat, goat, fox, European hedgehog, lesser hedgehog tenrec, or chicken ACE2. In total, 400, 200, 100, 50, and 25 nM of SARS-CoV-2 RBD, SARS-CoV RBD or SARS-CoV-2 NTD protein were used to monkey, mouse, rat, cat, bovine, horse, sheep, rabbit, raccoon dog, or hACE2. In total, 800, 400, 200, 100, and 50 nM of SARS-CoV-2 RBD, SARS-CoV RBD, or SARS-CoV-2 NTD protein were used to Malayan pangolin, wild Bactrian camel or alpaca ACE2. In total, 1600, 800, 400, 200, and 100 nM of SARS-CoV-2 RBD, SARS-CoV RBD or SARS-CoV-2 NTD protein were used to little brown bat or fulvous fruit bat ACE2. The anti-mFc antibody was regenerated with 10 mM Glycine-HCl (pH 1.7). The equilibrium dissociation constants (*K*_D_) of each pair of interaction were calculated using BIAcore^®^ 8K Evaluation Software (GE Healthcare) by fitting to a 1:1 Langmuir binding model. The supernatant containing hACE2-mFc protein was used as positive control.

### Pseudovirus transduction

Pseudotyped SARS-CoV-2 particles were obtained from National Institutes for Food and Drug Control of China. Pseudotyped SARS-CoV particles were produced in HEK293T cells as previously described^38^. In brief, cells were co-transfected with pNL4-3.luc.R-E- and pCAGGS-SARS-CoV-S plasmids with a 1:2 ratio. In total, 6 h later, the cell culture was replaced with fresh DMEM. In total, 48 h later, the supernatant containing the pseudotyped SARS-CoV were harvested, aliquoted and stored at −80 °C until use.

BHK21 cells were transfected with each of the 27 pEGFP-N1-ACE2s plasmids. 24 h later, eGFP-positive cells were sorted, reseeded in 96-well plates at 4 × 10^4^ cells/well and cultivated for another 24 h. The BHK21 cells were washed with PBS before the addition of the supernatant containing pseudovirus particles. Cells were lysed using the lysis buffer in the Luciferase Assay Systems (Promega) at 24 h post infection. In total, 10 μL of lysis supernatant was reacted with 50 μL of luciferase assay substrate and the luciferase activity was determined using a GloMax 96 Microplate luminometer (Promega). The BHK21 cells transfected with pEGFP-N1-hACE2 were used as positive control.

### Cryo-EM sample preparation, data collection, image processing, and model fitting

The complex protein of the cACE2 and the SARS-CoV-2 RBD (~0.2 mg/mL) was placed on a glow-discharged home-made graphene grid (Quantifiol Au 1.2/1.3, 300 mesh), stood for 10 s, blotted for 0.5 s with filter paper, and then the grid was plunged into liquid ethane using a FEI Vitrobot Mark IV.

The cryo-specimens were loaded on a 300 kV Titan Krios transmission electron microscope equipped with a GIF-Quantum energy filter and a Gatan K3 direct electron detector. Images were captured after 1.68 s exposure at a normal magnification of 130k and an electron dose rate of ~12.9 e^−^ pixel^−1^ s^−1^ using the counting mode, which resulted in a total dose of ~50 e^−^ Å^−2^ fractionated into 32 movie frames. The final defocus range of the datasets was roughly −1.8 to −2.2 μm.

The raw dose-fractionated images stacks were 3× Fourier binned, aligned, dose-weighted and summed using MotionCor2^39^. The initial contrast transfer function (CTF) parameters were estimated with CTFFIND4^40^. Then, 1551 good micrographs were manually selected from 1748 raw micrographs based on the Thon ring. All of the subsequent image processing and reconstruction were performed using Relion-3.1^41^. Briefly, a set of ~5000 particles was manually picked and subjected to 2D classification to generate templates for reference-based particle picking. A total of 1,500,357 automatically picked particles were extracted with a box size of 160 pixels and rescaled to 80 pixels in Relion-3.1 for the following 2D and 3D classification. One round of reference-free 2D classification was performed to remove the heterogeneous particles. A clean dataset with 837,848 particles from good 2D classes was selected and subjected to a second round 3D classification. After the second round of 3D classification, the predominant class containing a subset of 195,370 best particles shows the best structural features and the highest accuracy of particle alignment. The coordinates of these particles were exported in order to extract the full-size images for final reconstruction. The resulting density map at a resolution of 3 Å was determined by the Fourier shell correlation with a cutoff value of 0.143.

For the model of the cACE2 and SARS-CoV-2 RBD complex, the atomic model of hACE2 with the SARS-CoV-2 RBD (PBD 6LZG) was fit into the electron density map using Chimera^42^. The initial structure model was refined against the cryo-EM density map in real space using Phenix^43^ with secondary structure restraints. Automatic real-space and reciprocal-space refinements were performed using COOT^44^, and the stereochemical quality of the final model was assessed by MolProbity^45^.

## Supplementary information

Supplementary Information

## Data Availability

Cryo-EM map has been deposited in the Electron Microscopy Data Bank under accession code: EMD-30305.

## Supplementary information

**Supplementary Information** accompanies the paper at (10.1038/s41421-020-00210-9).
