# Supplementary material for: Broad host range of SARS-CoV-2 and the molecular basis for SARS-CoV-2 binding to cat ACE2
Source: Cell Discov. 2020 Sep 29;6:68. doi: 10.1038/s41421-020-00210-9 (PMC7526519; doi:10.1038/s41421-020-00210-9)
Supplement: Supplementary file 1 — Supplementary Information [file 41421_2020_210_MOESM1_ESM.pdf]

Supplementary information

Figure legends

Fig. S1 Sequences alignment of 27 ACE2s.

Position 83 was indicated in blue triangle. The sequence alignment was generated by ESPript.

Fig. S2 Conserved residues F28 and D355 contributed interactions for binding to SARS-CoV-2 RBD.

(a-b) Residues F28 and Y83 on cACE2 (a) or hACE2 (b). (c) Residue D355 of cACE2 involved interaction in cACE2 and SARS-CoV-2 RBD complex. (d) Residue D355 of hACE2 involved interaction in hACE2 and SARS-CoV-2 RBD complex. cACE2 and SARS-CoV-2 RBD bound to cACE2 were colored in palecyan and lightpink, respectively. The complex of hACE2 and the SARS-CoV-2 RBD was shown in gray. H-bonds were shown as dotted lines with a cutoff of 3.3 Å.

Fig. S3 Electrostatic surface views of SARS-CoV-2 RBD, hACE2-WT, and hACE2 with E37Q substitution.

(a) Electrostatic surface view of SARS-CoV-2 RBD. Residue Y505 was circled with dotted ellipse. (b) Electrostatic surface view of hACE2-WT. Residue E37 was circled with dotted ellipse. (c) Electrostatic surface view of hACE2-E37Q. Residue Q37 was circled with dotted ellipse.

Fig. S4 Binding of SARS-CoV-2 RBD or SARS-CoV RBD to rat and greater horseshoe bat ACE2s with the depletion of potential glycosylation at N82 by flow cytometry.

HEK293T cells transfected with pEGFP-N1-rat ACE2 or greater horseshoe bat ACE2, or the mutants containing N82M were incubated with His-tagged SARS-CoV-2 RBD or SARS-CoV RBD protein. Anti-His/APC antibody was used to detect the His-tagged protein binding to the cells. MERS-CoV RBD was used as the negative control.

Fig. S5 Transduction efficiency of SARS-CoV-2 pseudovirus mediated by civet ACE2.

The transduction efficiencies of three independent experiments of SARS-CoV-2 pseudovirus mediated by civet ACE2 were normalized to hACE2. The experiment was performed three times. Each histogram indicated the mean ± SD of three replicates in each experiment, with the original data displayed in the table. The values in parentheses represented transduction efficiencies of civet ACE2 normalized to the hACE2. The heatmap in Figure 3c was generated based on the data of Experiment 2, as highlighted by magenta rectangle.

Fig. S1

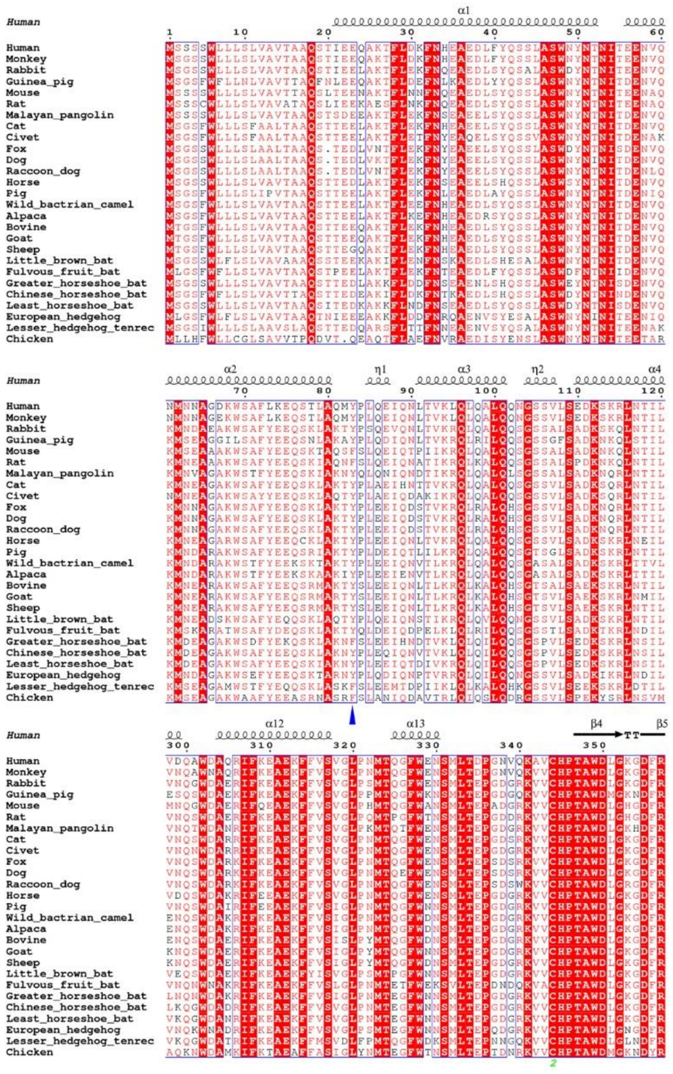

Fig. S2

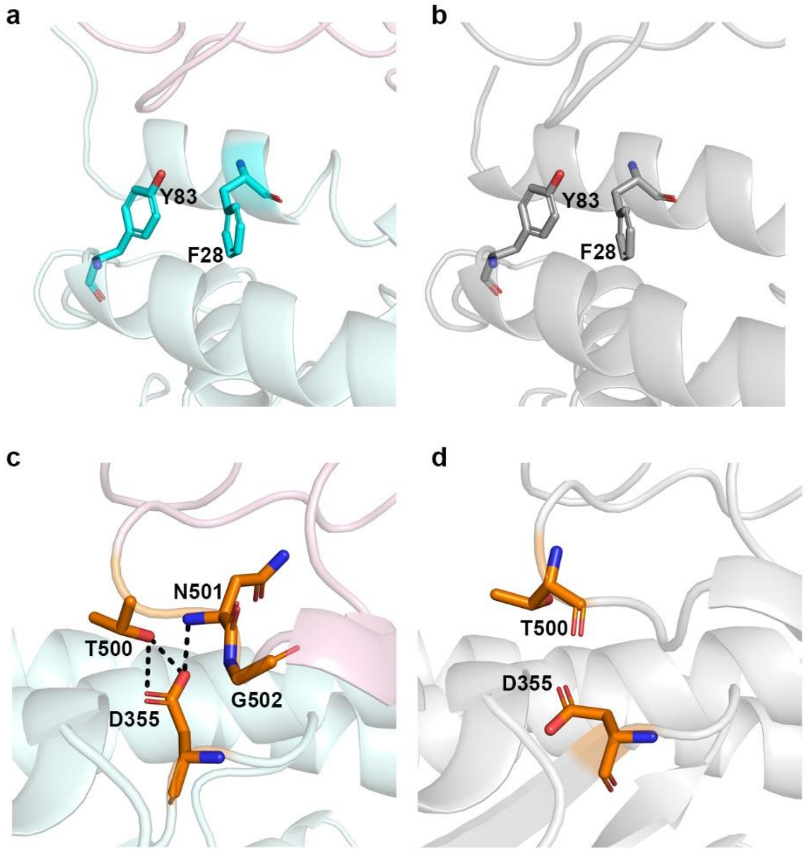

Fig. S3

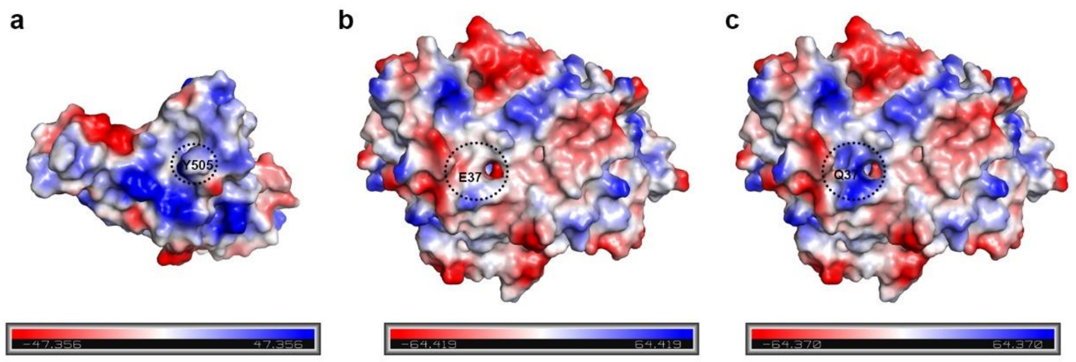

Fig. S4

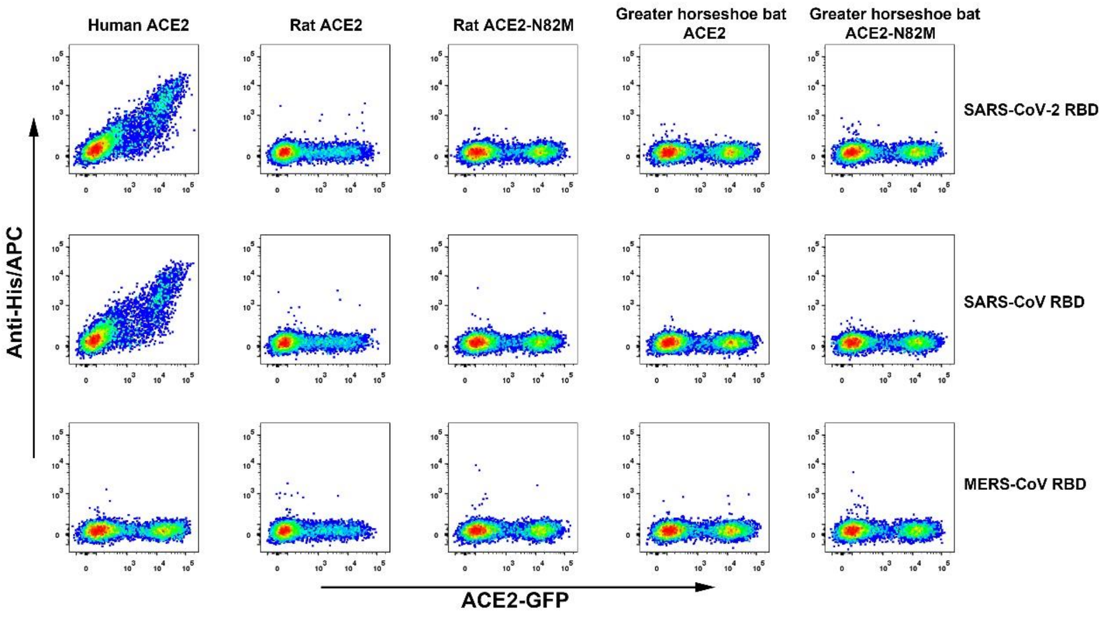

Human ACE2
Rat ACE2
Rat ACE2-N82M
Greater horseshoe bat ACE2
Greater horseshoe bat ACE2-N82M
SARS-CoV-2 RBD
SARS-CoV RBD
MERS-CoV RBD
Anti-His/APC
ACE2-GFP

Fig. S5

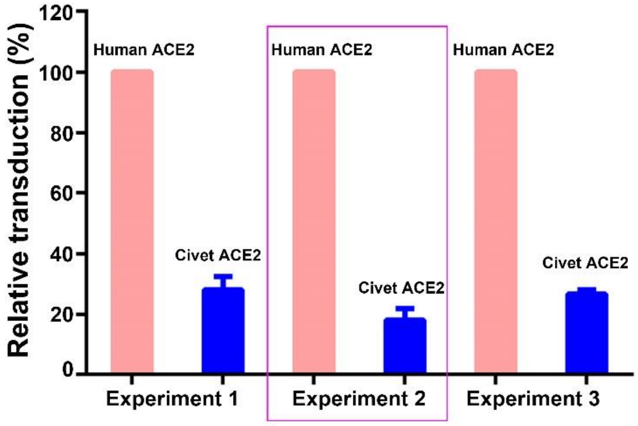

	Experiment 1	Experiment 2	Experiment 3
Human ACE2	2022696	2557513	2022696
	1027161	3570917	1414509
	1414509	1971749	
Civet ACE2	364717 (24.5%)	508436 (18.8%)	473867 (27.6%)
	490274 (32.9%)	371932 (13.8%)	443644 (25.8%)
	392240 (26.4%)	578086 (21.4%)	

Table S1. The accession numbers of 27 ACE2s

ACE2	Accession number
Human ACE2	BAJ21180
Monkey ACE2	A0A2K5X283
Rabbit ACE2	G1TEF4
Guinea pig ACE2	H0VSF6
Mouse ACE2	Q8R0I0
Rat ACE2	Q5EGZ1
Malayan pangolin ACE2	XP_017505746
Cat ACE2	Q56H28
Civet ACE2	Q56NL1.1
Fox ACE2	XP_025842512.1
Dog ACE2	J9P7Y2
Raccoon dog ACE2	ABW16956.1
Horse ACE2	F6V9L3
Pig ACE2	A0A220QT48
wild Bactrian camel ACE2	XP_006194263.1
Alpaca ACE2	XP_006212709.1
Bovine ACE2	Q58DD0
Goat ACE2	XP_005701129.2
Sheep ACE2	W5PSB6
Little brown bat ACE2	G1PXH7
Fulvous fruit bat ACE2	D8WU01
Greater horseshoe bat ACE2	B6ZGN7
Chinese horseshoe bat ACE2	E2DHI4
Least horseshoe bat ACE2	E2DHI9
European hedgehog ACE2	XP_007538670.1
Lesser hedgehog tenrec ACE2	XP_004710002.1
Chicken ACE2	F1NHR4
